# Water entry and exit in nature: review

**DOI:** 10.1098/rsfs.2024.0055

**Published:** 2025-05-16

**Authors:** Sunghwan Jung

**Affiliations:** ^1^ Department of Biological and Environmental Engineering, Cornell University, Ithaca, NY, USA

**Keywords:** diving, jumping, slamming, animals, water entry, water exit

## Abstract

Aquatic animals that live in water often leap out to catch prey in the air, while terrestrial and aerial animals dive into water to hunt aquatic animals. Some animals locomote on the water surface or lap water by repeated slamming and water-exiting motions. These dynamic interactions with the water–air interface have similarities in engineering, where water entry and exit problems play crucial roles in an object crossing the interface in industrial and physical systems. This review examines the physics of water entry and exit in biological systems through fluid mechanics principles originally developed for engineering applications. By identifying common governing forces, we aim to establish connections between biological strategies and engineering solutions, potentially leading innovations in bio-inspired technology.

## 1. Introduction

**Figure 1.**
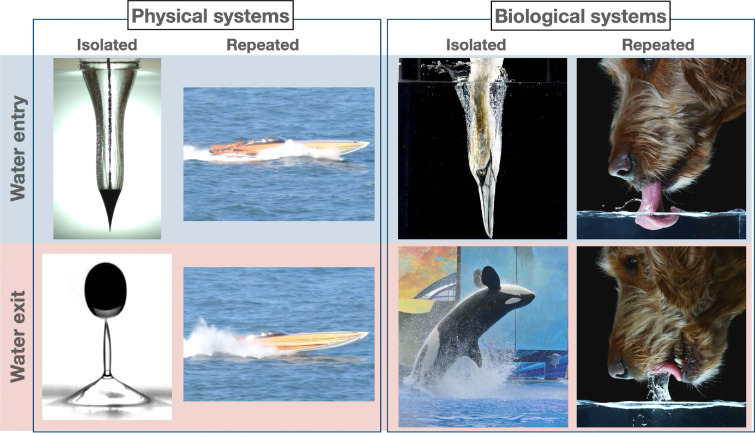
Examples of water entry and exit in physical (engineered) and biological systems: isolated water entry [1], water exit [2], repeated water entry and exit [3] in physical systems, and isolated water entry [4], water exit [5], repeated water entry and exit [6] in biological systems.

In nature, water serves as both a habitat and a medium for movement, enabling diverse organisms to transition between air and water for foraging, escape and locomotion. Animals dive into water, leap out of it, or move repeatedly between water and air (figure 1). The importance of these phenomena extends beyond biology and into engineering fields such as naval engineering and others. Ships, submarines and amphibious vehicles face similar forces and constraints when they interact with water, which has a long history in fluid mechanics.

This article reviews the dynamics of water entry and exit, primarily in biological systems, through the lens of fluid mechanics principles originally developed for engineering applications. We discuss the force balance and governing equations for objects entering and exiting water in §2. Section 3 presents various examples of animals engaging in water entry, water exit and repeated transitions between the two. Section 4 discusses engineering systems designed for water entry and exit.

## 2. Mechanics in water entry and exit

### 2.1. Force balance

**Figure 2.**
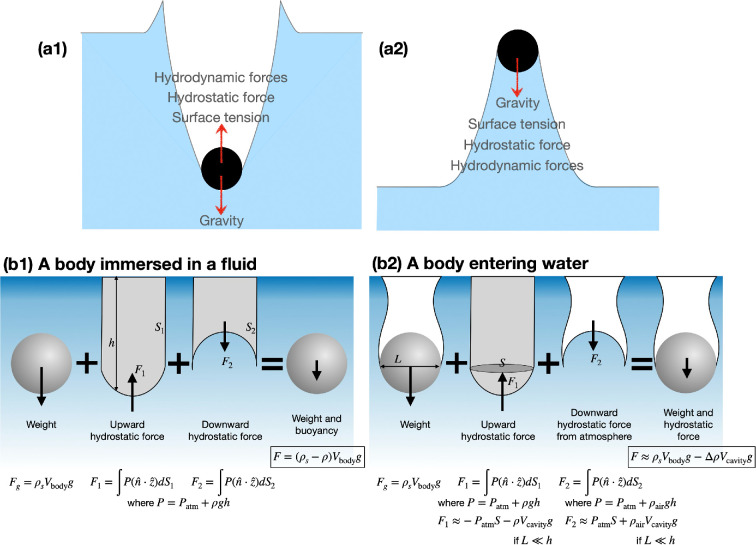
(a) Forces acting on a body during water entry and exit. (b) Gravitational and hydrostatic forces for a body fully immersed in a fluid versus a body entering water.

When an object impacts water, the object experiences several forces that govern its motion. These forces include surface tension, hydrodynamic lift/drag and gravity, as well as hydrostatic and added mass effects as shown in figure 2. Most forces described below have been discussed in the context of water entry extensively, while forces involved in water exit remain understudied.

When an object enters or exits across water, it displaces or carries some fluid along its path. The object not only has to overcome its own resistance but also the inertia of the surrounding water that it displaces. This displaced fluid virtually adds to the mass of the object, making it behave as if it were heavier than before the impact. This phenomenon is known as the ‘added mass’ effect. It significantly affects the dynamics of the object’s motion, during an object crossing two different media [7–9] or during rapid accelerations or decelerations in a uniform fluid medium (as a traditional example in Article 69 in [10]). Accounting for the added mass is crucial in understanding the impact force acting on biological organisms or in the design of submarines, ships and other marine vehicles to predict their behaviour accurately.

When an object contacts an interface, surface tension is dominant over inertia and gravity particularly for objects that are small, lightweight and/or slow moving [11]. Surface tension acts to minimize the surface area of a liquid, which typically keeps large water bodies relatively flat. When small insects interact with the water surface, the wettability of their bodies induces meniscus formation, creating curved surfaces that influence their locomotion. For larger objects, like ships or submarines, surface tension is less significant compared to other forces, but it can still influence the initial stages of water entry, affecting the splash and the formation of air cavities [12,13].

As an object moves through the water, it experiences hydrodynamic drag, which is the resistance the water exerts on the object’s motion. Drag arises from both viscous forces between the object’s surface and the water (skin friction) and the pressure difference between the front and rear of the object (pressure drag) [14–16]. Hydrodynamic drag depends on factors such as the object’s shape, size, velocity and surface roughness. Streamlined body shapes, achieved by animals folding their appendages and minimizing resistance, help reduce drag and enhance efficiency in water. In high-speed water-entry scenarios, such as diving dolphins or birds, reducing hydrodynamic drag is critical for maintaining speed and stability.

Once submerged, the object experiences buoyancy, which is the upward force exerted by the displaced water on the object [15]. Buoyancy is governed by Archimedes’ principle, which states that an object floated/immersed in a fluid experiences a buoyant force equal to the weight of the fluid it displaces. If the object’s density is less than that of water, it will float; if it is denser, it will sink. Buoyancy arises from the pressure difference across an object’s submerged surfaces, governed by hydrostatic pressure. For a fully submerged object, the net buoyant force results from the integral of hydrostatic pressure acting on its surface. However, for a water-entering object particularly with an attached air cavity, the force distribution is slightly complicated. The upper portion of the object may be exposed to atmospheric pressure, while the lower portion experiences hydrostatic pressure with depth. This asymmetry makes the resisting force stronger in a water-entering object. This asymmetry enhances the resisting force during water entry. To accurately describe this force balance, hydrostatic pressure depending on the depth should be integrated only over the water-contact area rather than the entire surface of the object. A useful approximation is to consider the hydrostatic pressure acting on the lower surface of the object below its equator (which further approximates the buoyancy of the air cavity [17]) with the object’s weight. This hydrostatic pressure difference across the water-entering body generates an upward force due to the high hydrostatic pressure underneath the body. However, if the object is moving rapidly during water entry, the hydrostatic effect becomes negligible due to its high inertia. Furthermore, the air cavity pinches off early during the water-entry process, limiting the influence of hydrostatic pressure on the overall dynamics of the water entry.

In high-speed water-entry scenarios, cavitation may also occur. This phenomenon happens when the pressure around the animal drops below the vapour pressure of the water, leading to the formation of vapour bubbles. Cavitation can cause a sudden increase in drag and potentially damage surfaces due to the collapse of vapour bubbles, which can create intense localized forces. Cavitation is particularly relevant in the design of high-speed propellers, torpedoes and submarines however, it was not clearly observed on animal dives. Cavitation occurs when the local pressure around a moving object drops below the vapour pressure of water, leading to bubble formation. For cavitation to occur, the dynamic pressure $\frac{1}{2}\rho V^{2}$ must exceed the pressure difference between the reference pressure (about 101.3 kPa = 1 atm) and the vapour pressure of water (about 2.3 kPa at 25°C). Based on this criterion, the velocity required to induce cavitation in water is approximately 14.1 m s^−1^. Several plunge-diving birds, including terns, pelicans, boobies and gannets, are capable of reaching these speeds upon impact, suggesting that cavitation bubbles could theoretically form around their beaks or bodies during high-speed entry.

### 2.2. Non-dimensional numbers and governing equation

To understand the forces acting on objects, non-dimensional numbers are widely used. Non-dimensional numbers simplify the analysis of complex hydrodynamic interactions by quantifying the relative influence of competing forces, allowing for direct comparisons of behaviours across a wide range of organisms and conditions. Each non-dimensional number helps to simplify the analysis of fluid–animal interactions.

The Reynolds number is one of the most fundamental non-dimensional numbers in fluid dynamics. It compares the inertial forces to viscous forces in a fluid, indicating whether the flow is laminar or turbulent. For an object moving through water, the Reynolds number is given by


(2.1) $$\text{R}\text{e}=\frac{\rho VL}{\mu }\propto \frac{\rho fM^{2/3}}{\mu }\;,$$

where ρ is the fluid density, V is the object’s velocity relative to the fluid, L is a characteristic length of the object (e.g. diameter or length of an animal) and μ is the dynamic viscosity of the fluid. Table 1 highlights differences in surface tension and viscosity between natural and industrial fluids, which might be useful in biological versus engineered contexts. If you consider animals moving in fluid, then the animal’s frequency, f, and body mass, M, can be used to define the Reynolds number. In allometry, an animal’s mass is proportional to the cube of its length [28]. Additionally, the object’s velocity V scales as fL. In the simplest model, the swimming or flying speed of steady swimming or flying animals, U, scales with its frequency as f∼U/L [29–31].

**Table 1.** Surface tension and dynamic viscosity of various fluids.

fluids	surface tension (mN m^−1^)	dynamic viscosity (mPa s^−1^)
pure water	71.99 at 25°C [18]	1.00 at 20°C [18]
tap water	72.40 at 25°C [19]	0.96 at 21°C [20]
sea water	72.26 at 30°C and salinity 35.20 [21]	~0.97 at 25°C and salinity 35 [22]
glycerol	~63.4 at 20°C [23]	1500 at 20°C [23]
ethanol	21.97 at 25°C [18]	1.1 at 25°C [18]
acetone	23.46 at 25°C [18]	0.31 at 25°C [18]
mineral oil	26.1−29.3 at 25°C [24]	9.85−9.89 at 25°C [25]
olive oil	33.00−33.06 at 20°C [26]	89.6 at 20°C [27]

The Reynolds number is crucial when assessing the relative strength of inertia to viscous stress and how the fluid behaves around animals [32–35]. The allometry and scaling of swimming animals in a uniform medium (most likely water) are well studied [30,31,36,37]. In the case of high-speed water entry, such as bird and dolphin diving and projectile penetration, the Reynolds number is typically large, indicating turbulent flow around the body [31].

The Froude number compares the inertial to gravitational forces, particularly relevant in determining an object’s gravitational effect and behaviour on or near a fluid’s surface. The Froude number is given by


(2.2) $$\text{Fr}=\frac{V}{\sqrt{gL}}\propto \frac{fM^{1/6}}{\sqrt{g}},$$

where g is the gravitational acceleration.

For surface-moving objects like boats, hydrofoils or animals locomoting on water, the Froude number determines whether the object is primarily driven by gravity or inertia. At low Froude numbers, the gravitational force dominates and the object floats or moves slowly across the surface. A high Froude number allows animals to defy gravity using inertia, which benefits flying animals taking off from the water (e.g. grebes, loons and ducks) and terrestrial/amphibian animals skittering on the water surface (e.g. frogs [38], geckos and basilisk lizards [39]). Another interesting example is about skipping stones providing a fascinating phenomenon of high‑Froude-number dynamics [40,41]. When a stone is thrown with sufficient speed, the inertial forces are balanced by buoyancy and drag momentarily, allowing the stone to rebound off the water’s surface. Likewise, the Froude number plays a crucial role in hydrodynamic interactions with the free surface, helping to understand body movement.

The Weber number quantifies the relative importance of inertial to surface tension forces, especially during low-speed water entry or exit when surface tension becomes significant. It is defined as


(2.3) $$\text{We}=\frac{\rho V^{2}L}{\sigma }\propto \frac{\rho f^{2}M}{\sigma },$$

where σ is the surface tension of the water. In table 1, the surface tension values of various natural and industrial fluids are summarized.

In scenarios where the Weber number is low, surface tension dominates, as seen in the entry of small objects like water droplets or insects. For larger objects, such as diving birds or dolphins, surface tension is less critical compared to other forces like drag or inertia, and the Weber number is correspondingly higher. However, for small and lightweight objects but with high impact velocity (e.g. droplets hitting a water surface), surface tension influences small-scale features like splash formation and air cavities formed around the object. In general, a higher surface tension leads to a flatter interface and increased resistance to deformation, resulting in less pronounced splashing. On the other hand, a lower surface tension allows for larger splash formation. This capillary (i.e. surface tension) effect is particularly strong for small-scale organisms, such as diving and water-jumping insects, where surface tension significantly influences entry dynamics.

The Bond number is a dimensionless parameter used to estimate the relative strength of gravitational or buoyant force compared to surface tension force as


(2.4) $$\text{Bo}=\frac{\rho gL^{2}}{\sigma }\equiv \frac{\text{We}}{{\text{Fr}}^{2}}\propto \frac{\rho gM^{2/3}}{\sigma }\;.$$

It can also be expressed as the ratio of the Weber number to the square of the Froude number. Some variations of the Bond number are used in different contexts. The first variation incorporates the density difference between the body density, $\rho_{s}$, and the fluid density, ρ. In that case, the Bond number defining $(\rho_{s}-\rho )gL^{2}/\sigma$ can include the body weight and buoyancy relative to the surface tension, which is useful when analysing floating or submerged objects. The second variation is to use the body acceleration, a, instead of the gravitational acceleration as ${\text{Bo}}^{\left(a\right)}=\rho aL^{2}/\sigma$. This modification is crucial for characterizing fluid dynamics in scenarios where the object’s acceleration exceeds gravitational acceleration, such as in the cyclic motions of animals. For example, cats and dogs lap water with their tongues at accelerations greater than g, in which the tongue acceleration is important in fluid motions [6,42,43].

The Strouhal number is a dimensionless quantity that describes the relationship between unsteady and steady forces in oscillatory or periodic flows. It is particularly relevant in understanding vortex shedding, oscillations of objects in a fluid and propulsion mechanisms in swimming and flying organisms. The Strouhal number is defined as


(2.5) $$\text{St}=\frac{fL}{U},$$

where f is the frequency of oscillation or vortex shedding. It is worth noting that the Strouhal number tends to remain constant when the condition U=fL is met; the condition is commonly observed in the swimming and flying of animals at certain Reynolds numbers. The Strouhal number is crucial in contexts where unsteady forces play a significant role. For animals, the Strouhal number is commonly used to analyse cyclic appendage motions in locomotion. Certain animals, such as basilisk lizards, water striders and some birds, generate thrust forces on the water surface during movement. The Strouhal number was also used to characterize the efficiency of these repeated motions in facilitating motion across or through the water [36,44]. Here, the Strouhal number becomes constant if one considers that the flow velocity, U, scales as the animal’s flapping frequency times its characteristic length [29–31].

When a fluid flows past a bluff body, alternating vortices are shed from opposite sides of the body, creating a characteristic oscillatory flow and inducing the vibration of the body [45,46]. The Strouhal number helps predict the vortex shedding frequency, which is important to understand the force generated by animals. In swimming and flying, animals generate oscillatory motions to propel themselves. For instance, the flapping of wings in birds or fins in fish can be characterized by the Strouhal number, which is optimized within a specific range (0.2 < St < 0.4) for efficient propulsion [33,44]. The Strouhal number determines a transition between steady and unsteady flow phenomena, e.g. oscillatory motion in fluids.

The added mass coefficient is a non-dimensional number representing the proportion of the mass of the surrounding fluid that contributes to the inertia of the object during motions through the fluid. It is typically expressed as


(2.6) $$C_{m}=\frac{m_{added}}{M},$$

where $m_{\mathrm{added}}$ is the added mass (the mass of the displaced fluid) and M is the mass of the object. The added mass coefficient becomes particularly important while an object penetrating another fluid, such as water entry and exit. This effect is significant in engineering applications like submarine and ship design, where added mass affects the object’s manoeuvrability and dynamic stability.

**Figure 3.**
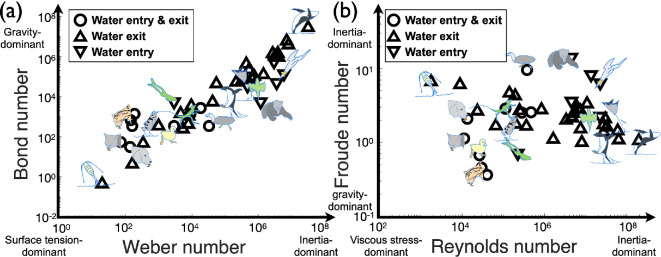
(a) Bond versus Weber numbers and (b) Froude versus Reynolds numbers of animals crossing the interface based on data listed in table 2. A clear power‐law relationship is observed between the Bond and Weber numbers. In contrast, the Froude number varies within a narrow range of approximately 1.5 orders of magnitude, while the Reynolds number spans nearly five orders of magnitude. Most leaping and water-running animals exhibit Froude numbers between 1 and 10, maintaining controlled movement without excessive overshooting, despite significant variations in their Reynolds numbers.

Figure 3 illustrates the relationship between various dimensionless numbers characterizing the water interface-crossing behaviours of different animals. In figure 3a, the Bond number is plotted against the Weber number, showing a positive correlation, while in figure 3b, the Froude number is plotted against the Reynolds number, where the Froude number plateaus with increasing Reynolds numbers. It is worth noting that the magnitudes of these non-dimensional numbers are not directly related to the animals’ weight but rather to their behaviours. For instance, animals engaging in lapping behaviours, such as cats and dogs, are located in regions of low Bond, Weber, Reynolds and Froude numbers. In contrast, high-speed divers, such as birds performing plunge-diving, are positioned in areas of higher values for these numbers. Intermediate behaviours, like skittering or planing, locate the middle range. These distinct clusters reflect the diversity in physiological adaptations and behavioural purposes, which naturally lead to a lack of clear linear correlations among the dimensionless numbers.

**Table 2.** Physical kinematics and non-dimensional numbers for a few selected species, categorized by behaviour (see Data accessibility). The characteristic length is chosen based on body length or width for skimming and diving animals, and tongue size for drinking animals.

species	behaviour	freq.	length	vel.	We	Bo	St	Re	Fr	ref.
		(Hz)	(m)	(m s^−1^)	$\frac{\rho V^{2}L}{\sigma }$	$\frac{\rho gL^{2}}{\sigma }$	$\frac{fL}{U}$	$\frac{\rho VL}{\mu }$	$\frac{V}{\sqrt{gL}}$	
lizard	water running	8.00	0.10	2.50	$8.93\times 10^{3}$	$1.36\times 10^{3}$	$3.20\times 10^{-1}$	$2.80\times 10^{5}$	2.53	[39]
streamer	skimming	2.00	0.05	6.67	$3.21\times 10^{4}$	$3.39\times 10^{2}$	$1.49\times 10^{-2}$	$3.75\times 10^{5}$	9.57	[47]
duckling	paddling	7.12	0.05	0.47	$1.58\times 10^{2}$	$3.39\times 10^{2}$	$7.45\times 10^{-1}$	$2.63\times 10^{4}$	0.67	[48]
dog	lapping	3.00	0.015	0.80	$1.37\times 10^{2}$	$3.05\times 10^{1}$	$5.63\times 10^{-2}$	$1.34\times 10^{4}$	2.09	[6]
cat	lapping	3.50	0.020	0.50	$7.14\times 10^{1}$	$5.43\times 10^{1}$	$1.40\times 10^{-1}$	$1.12\times 10^{4}$	1.13	[49]
lion	lapping	1.80	0.10	0.36	$1.85\times 10^{2}$	$1.36\times 10^{3}$	$5.00\times 10^{-1}$	$4.03\times 10^{4}$	0.36	[49]
ocelot	lapping	2.70	0.07	0.38	$1.43\times 10^{2}$	$6.65\times 10^{2}$	$5.00\times 10^{-1}$	$2.96\times 10^{4}$	0.46	[49]
pelican	plunge diving	0	2.00	10.00	$2.86\times 10^{6}$	$5.43\times 10^{5}$	0	$2.24\times 10^{7}$	2.26	[50]
gannet	plunge diving	0	1.00	20.00	$5.71\times 10^{6}$	$1.36\times 10^{5}$	0	$2.24\times 10^{7}$	6.39	[4]
kingfisher	plunge diving	0	0.50	8.00	$4.57\times 10^{5}$	$3.39\times 10^{4}$	0	$4.48\times 10^{6}$	3.61	[51]
frog	diving	0	0.20	1.00	$2.86\times 10^{3}$	$5.43\times 10^{3}$	0	$2.24\times 10^{5}$	0.71	[52]
bear	diving	0	0.20	20.00	$1.14\times 10^{6}$	$5.43\times 10^{3}$	0	$4.48\times 10^{6}$	14.3	[53,54]
osprey	plunge diving	0	0.60	20.00	$3.43\times 10^{6}$	$4.89\times 10^{4}$	0	$1.34\times 10^{7}$	8.25	[55]

### 2.3. Governing equation

**Figure 4.**
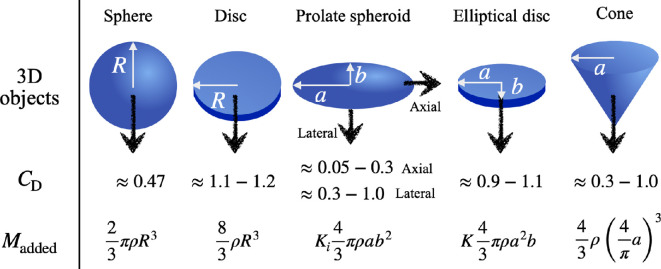
Drag coefficient [16] and added mass [31] of various shaped objects.


The forces described above can be written in a differential equation form. The governing equation reads


(2.7) $$\frac{\text{d}}{\text{d}t}[(M+m_{added})\text{u}]≃\rho_{s}V_{body}g\hat{z}-\Delta \rho V_{cavity}g\hat{z}-\frac{1}{2}\rho C_{D}S|\text{u}{|}^{2}\hat{t}-\frac{1}{2}\rho C_{L}S|\text{u}{|}^{2}\hat{n}-\sigma L\hat{n}+{\text{F}}_{ext},$$

where $\rho_{s}$ is the body density, Δρ is the density difference between the lower and upper fluids, $V_{\mathrm{body}}(\equiv SL)$ and $V_{\mathrm{cavity}}(\approx S\times h$; h is the depth of the body) are the volumes of the body and the fluid cavity entrained by the impacting body, L is the line integral of the vertical component of the surface tension along the contact line on the body, and $\hat{n}$ and $\hat{t}$ are the normal and tangential direction to the body velocity u. Here, $C_{D}$ and $C_{L}$ are the drag and lift coefficients (see the list of drag coefficients of various shapes in figure 4). This equation represents the balance of inertial forces, gravitational forces, hydrodynamic drag and lift, hydrostatic and surface tension forces, along with any external forces. The governing equation in the non-dimensional form becomes

(2.8) $$\text{St}\rho^{*}\frac{\text{d}}{\text{d}t^{*}}[(1+C_{m}){\text{u}}^{*}]≃\frac{1}{{\text{Fr}}^{2}}\left(\rho^{*}-\frac{\Delta \rho }{\rho }\frac{V_{cavity}}{V_{body}}\right)\hat{z}-\frac{C_{D}}{2}|{\text{u}}^{*}{|}^{2}\hat{t}-\frac{C_{L}}{2}|{\text{u}}^{*}{|}^{2}\hat{n}-\frac{1}{\text{We}}\frac{L\text{L}}{\text{S}}\hat{z}+\frac{{\text{F}}_{ext}}{\rho SV^{2}}\;,$$

where ${\text{u}}^{*}$ and $t^{*}$ are dimensionless velocity and time scales normalized by the characteristic velocity and the inverse frequency, and $\rho^{*}(\equiv \rho_{s}/\rho )$ is the density ratio between the body and fluid. For water-entry problems, this formula can be used directly without much modifications. However, for water-exit cases, a change in the entrained fluid volume can act as extra external resistance force as $-\text{d}(m_{f}\text{u})/\text{d}t$, where $m_{f}(t)$ is the mass of the entrained fluid ($\equiv \Delta \rho V_{\mathrm{cavity}}$) and z is the position of the object [5]. The reason that this term should be considered in water exit is that typically a lower fluid is heavier (e.g. water), and the entrained fluid mass may not be smaller than the body weight. In the case of water entry, the entrained upper fluid (e.g. air) is much lighter than the body weight. Hence, we can ignore this term in the water-entry problems. This governing equation along with non-dimensional numbers allows us to identify the relative strength of each term and ignore some terms for simplifications across various conditions.

### 2.4. Impact force

Impact force is a crucial factor in both biological and engineered water-entry systems. In biological contexts, a high-impact force can lead to injuries, such as neck injuries or bone/organ damages in animals. In engineered systems, impact forces must be carefully managed to prevent mechanical failure, structural deformation or electrical malfunctions in aerial–aquatic vehicles, naval operations and space capsule re-entry. For biological systems, at a given mass, the relative importance of different forces can be evaluated using the non-dimensional numbers introduced in equations (2.1)–(2.4). When inertia is dominant, the impact force becomes significant and cannot be ignored, as it scales similarly to inertial forces.

Two classical methods in water-entry studies are the von Karman method [8] and the Wagner method [7]. The von Karman method simplifies the problem by ignoring the local rise of water during impact, while the Wagner method accounts for this water rise but assumes a blunt body impact. Most theoretical studies of water-entry focus on two-dimensional (2D) vertical impacts of symmetric bodies, as this simplifies the analysis [7,9,31]. However, in real-world applications, three-dimensional flow effects can be significant.

When analysing the impact force acting on an object in two dimensions, complex analysis helps by interpreting the problem into a more mathematically manageable form using a complex potential. The force acting on the object can be calculated by integrating the pressure difference around the surface. The resulting force expression explains the added mass effect, with the force depending on the time derivative of the added mass and the velocity. The impact force simplifies to $F_{impact}=-\frac{V}{2}\frac{\text{d}}{\text{d}t}\left(\pi \rho c^{2}\right)-\frac{1}{2}\pi \rho c^{2}\frac{\text{d}V}{\text{d}t}$, where c is the half-width of the plate and $\rho \pi c^{2}$ is the added mass of a 2D plate. This approach can be generalized to different body shapes, allowing the force calculation for various objects moving in a fluid.

The impact force for an object entering a fluid at constant velocity can be expressed as


(2.9) $$F_{impact}=\frac{V}{2}\frac{\text{d}m_{added}}{\text{d}t}=\frac{1}{2}\frac{\text{d}m_{added}}{\text{d}\zeta }V^{2}\;.$$

Here, the time, t, can be rewritten as ζ/V where ζ is the depth of the object in the fluid. As shown in figure 4, the added mass is proportional to $\rho L^{3}$, where L is the characteristic length scale of the object. Substituting this relationship, the impact force can be rewritten as


(2.10) $$F_{impact}∼\rho L^{2}\frac{\text{d}L}{\text{d}\zeta }V^{2}\;.$$

The body shape, specifically the variation of its radius with depth, plays a critical role in determining the magnitude and progression of the impact force.

**Figure 5.**
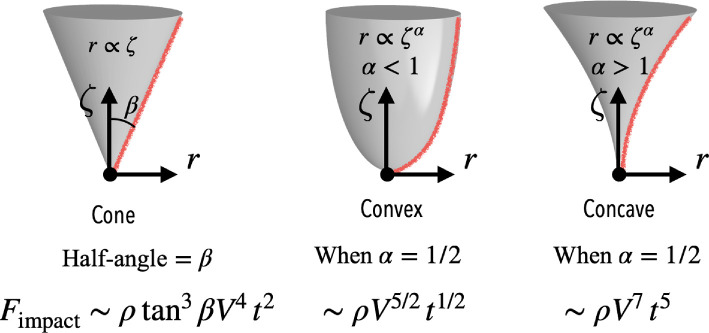
Impact forces of cone, convex and concave diving fronts. Further details, including force versus time plots, can be found in [56].

For axisymmetric bodies, three representative shapes can be considered to explore how the impact force evolves as shown in figure 5. The radius of the body, r, can be used as the characteristic length, L. The first case is a conical diving front, where the radius is linearly proportional to the depth (r∝ζ). For this shape, the impact force scales with the square of the depth and velocity, $\zeta^{2}V^{2}$. Assuming a constant impact velocity (ζ=Vt), this relationship transforms into $F_{\mathrm{impact}}\propto V^{4}t^{2}$, indicating a rapid growth in the impact force over time.

The second category is bodies with convex or concave diving fronts, where the radius is approximated as a power‐law relationship with depth, $r\propto \zeta^{\alpha }$. For convex shapes (0<α<1), the impact force scales as $F_{\mathrm{impact}}\propto \rho V^{3\alpha +1}t^{3\alpha -1}$. Similarly, for concave shapes (α>1), the same scaling laws apply but with different shapes determined by α. For instance, in the case of a quadratic convex diving front (α=1/2), the impact force becomes $F_{\mathrm{impact}}\propto \rho V^{5/2}t^{1/2}$. This result suggests a steep initial increase in force, followed by a more gradual rise as time progresses. For the case of a concave body with α=2, the impact force becomes $F_{\mathrm{impact}}\propto \rho V^{7}t^{5}$.

These results are consistent with physical intuition. A blunt body, such as one with a flat or rounded front, typically experiences a sharp spike in impact force at the moment of collision due to the rapid increase in the surface contact area. For diving animals, approximated as prolate spheroids, the impact force is estimated based on the cross-sectional area in contact with the fluid, with belly-first or head-first dives generating greater forces due to the larger surface area compared to arm-first dives [56].

## 3. Biological systems

### 3.1. Water entry by aerial and terrestrial animals

The mechanics of water entry involve a rapid transition from air to a much denser medium (e.g. fresh or sea water), where resistance increases dramatically. The key factors influencing successful water entry include animal’s shape, entry angle and speed.

**Figure 6.**
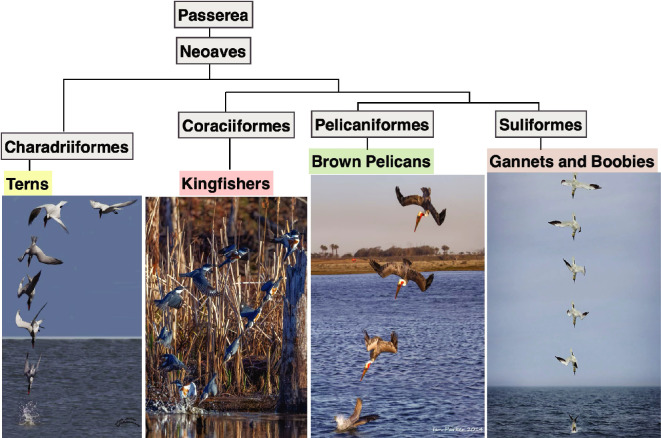
Plunge-diving birds: terns (image courtesy of Jack Sutton), kingfishers (image courtesy of Skikanth Boga), pelicans (image courtesy of Ian Parker) and gannets (image courtesy of Claire Marshall) from left to right. The phylogeny is based on [64].

Fish dwelling in water are a primary food source for many aerial bird species, making it essential for these birds to cross the water–air interface to forage. In general, birds have evolved diverse foraging strategies to access aquatic prey, including diving from high altitudes (plunge diving) and diving from the water surface (surface diving). These distinct behaviours are accompanied by specialized morphological adaptations, such as modifications to the beak [57], skull [58,59], neck [60] and feathers [61–63]. In particular, some seabirds (e.g. gannets, boobies, terns and brown pelicans) and freshwater birds (e.g. most kingfishers) are known for their regular plunge-diving behaviours (figure 6). Biologists have extensively studied the ecological and biomechanical aspects of plunge diving in birds. For instance, younger Sandwich terns exhibit lower fishing success rates than adults due to less experienced and optimized hunting behaviours [65]. Cape gannets showed that their high-speed dives are optimized for prey capture efficiency [66]. Northern gannets plunge as deep as 22 m as measured in [67]. White-chinned petrels dive at different depths depending on the location of food sources [68]. Escape responses of black mollies to predatory dives by pied kingfishers showed the dynamic predator–prey interactions at the air–water interface [69].

Beyond ecological factors, studies have also examined the biomechanics of diving. Australasian gannets preselect their dive profiles before impact, revealing distinct *U*- and *V*-shaped dive patterns based on prey location and showing that deeper *U*-shaped dives had higher prey capture success rates [70,71]. Northern gannets use underwater wingbeats to extend their diving depth and duration, emphasizing the role of propulsion beyond passive plunge momentum [72]. An unusual plunge-diving manoeuvre in brown pelicans involves an inverted entry and a split-S turn-like movement, suggesting potential benefits for prey capture success rate [73].

Plunge diving is the act of water entry from mid-air, which could be a dangerous activity when the impact force exceeds the body’s physiological capability to withstand. A few species of birds are capable of diving into water at a speed of more than 20 m s^−1^ [66,74]. Thus, their bodies must be able to cope with high mechanical stresses during impact [4]. Researchers have investigated this diving behaviour in terms of ecological factors (e.g. diving depth and prey species) and body-shape configurations (e.g. arrow-like body posture and diving angle and speed) [67,68,70,72,75]. For plunge-diving birds, the behaviours and morphology would have gone through a similar evolutionary adaptation to withstand the mechanical forces [4,76].

Previous work on plunge-diving birds involves studies on beak and skull morphology and studies on bio-inspired neck dynamics. Most studies focused on bird beak and skull shape without considering the anatomy and structure of the neck [4,76–79]. The underlying reason for focusing on beak–skull morphology is the impact force originating in the dive front (frontal shape). Recent studies have employed nonlinear modelling and experimental validation to analyse the structural integrity of diving systems [80,81]. These studies demonstrate that beak shape, joint flexibility, and material stiffness play pivotal roles in determining an organism’s ability to withstand impact [80–82].

Plunge-diving behaviour has repeatedly evolved in bird lineages that are distantly related in a phylogenetic tree. Studying these lineages revealed convergent morphological and anatomical (beak, skull and neck) features of kingfishers in adaptation to their behaviour or, alternatively, different anatomical solutions to optimal plunge diving [76,78]. Understanding the biomechanics of plunge diving is critical to clarifying how the impact forces impose structural limitations to the morphology of these birds.

On the other hand, terrestrial and amphibian animals often leap into water for hunting or escaping predators, demonstrating their remarkable ability to temporarily engage with aquatic environments. Since these animals primarily inhabit land, their behaviours are adapted to exploit aquatic resources or evade threats effectively. However, physiological adaptations may not be achieved since their water-related behaviours are occasional and context dependent.

Frogs are another group of terrestrial/amphibian animals that frequently leap into water to hunt. Many frog species spend a significant portion of their time near aquatic environments, where they can catch insect larvae, small fish and other invertebrates. Frogs often leap from land into water to catch prey or to escape from predators, using their powerful hind legs to propel themselves once submerged [52,83]. When frogs re-enter the water after a leap, they tuck their forelimbs close to their bodies, creating a streamlined shape that minimizes impact force by allowing fluid to flow smoothly around their heads.

**Figure 7.**
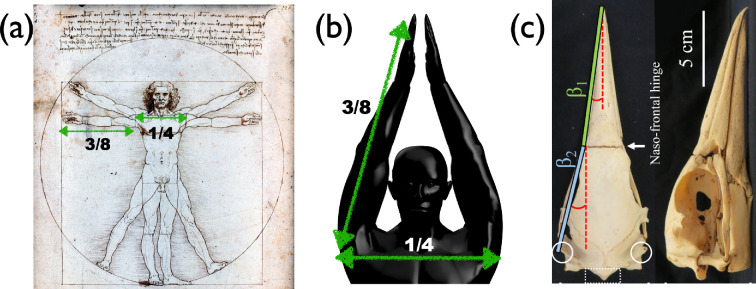
(a) Vitruvian Man by Leonardo da Vinci (image courtesy of Paris Orlando). (b) A possible arm angle of a human. (c) A beak and skull of gannet [4].

The angle of a cone-shaped diving front, as previously discussed, is a critical parameter in water-entry dynamics. In impact dynamics, engineers conventionally use the half-angle, which is half of the full cone angle. Plunge-diving birds, such as kingfishers, terns, pelicans, boobies and gannets, have evolved to possess beaks with smaller angles (e.g. approximately 10° for gannets) and/or a smooth transition from their beaks to their foreheads, optimizing their entry into water. However, detailed data and analysis are needed to confirm these evolutionary trends and their potential effects. Humans, on the other hand, are highly flexible and can adopt different shapes by changing their body posture when diving. According to previous studies [56], the impact force during water entry can be minimized if a human forms a wedge-like posture, commonly referred to as arm-first diving. While a certain entry posture can reduce impact force, competitive divers often use flat-hand entries to generate controlled air cavities, reducing splash [84,85]. The angle that a human can achieve in this posture can be estimated based on basic body geometry (figure 7). Leonardo da Vinci’s Vitruvian Man provides useful proportions: the shoulder width is about one-quarter of the body height, and the arm length is approximately three-eighths of the height. Using simple trigonometry, the half-angle of an arm-first diving posture can be calculated as roughly 20° ($≃\sin^{-1}\left((1/8)/(3/8)\right)$). Since the impact force is proportional to the cube of the half-angle, reducing the angle significantly decreases the force. However, even with this optimization, humans cannot match the efficiency of the specialized beak structures of diving birds, which have evolved to minimize their half-angle and, consequently, their impact force during water entry. The impact force on birds can be lower by a factor of 9 solely based on the angle alone compared to human diving. To fully estimate the total impact force, we need to account for cross-sectional area and diving velocity. Overall, plunge-diving birds experience much lower impact forces than humans not only due to their smaller beak angles but also due to their smaller cross-sectional areas.

### 3.2. Water exit by aquatic animals

**Figure 8.**
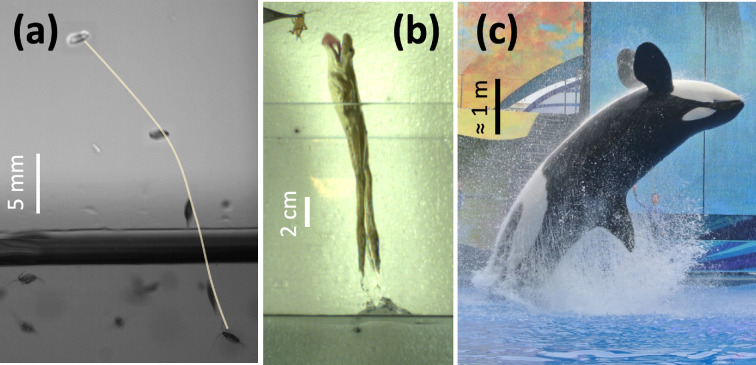
Various jumping animals: (a) copepod (image courtesy of Brad Gemmell), (b) frog (image courtesy of Talia Weiss and Jake Socha) and (c) orca [5].

Many animals leap out of water to escape from predators, to capture prey, breathe, communicate or even recreationally [5] as shown in figure 8. For example, copepods jump out of water when a predator (typically fish) approaches them from below [86,87]. Some frogs and fish are able to leap out of water to catch prey [52,88,89] or to escape from external stimuli [90–93] using pure hydrodynamic forces. Humans jump out as a recreational activity [94,95]. Larger animals, such as penguins [96,97], stingrays [98,99], dolphins [100–104], sharks [105] and whales [106], also leap out of water.

For successful water exit, the forces that push an animal out of the water must overcome the hydrodynamic resistance. This requires significant muscle strength and streamlined body shapes to minimize drag. A certain species of copepods (Labidoceraaestiva) are able to leap out more than 25 times their own body length [86,87], which is not an easy task due to their sub-capillary length scale. The capillary force is particularly strong for small organisms, making surface-breaking more difficult compared to larger animals with greater inertia. Trinidadian guppy is known to jump more than three times its body length with rapid fin undulations [93]. Archerfish kinematics show they can jump around twice body lengths [107], which shows strong thrust generation from undulations.

### 3.3. Combined water entry and exit

Most research has primarily focused on either water entry or water exit as a separate topic. However, many behaviours in nature (e.g. locomotion or water lapping) involve both water entry and exit processes in a periodic fashion.

**Figure 9.**
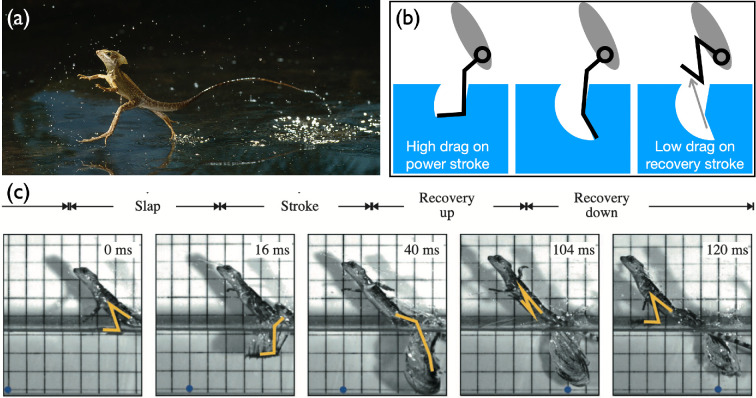
Water-running lizard. (a) Basilik running on water in nature (image courtesy of Stephen Dalton). (b) Schematic of a foot slamming and exiting out of the air cavity. (c) Different phases of a water-running lizard (image courtesy of Tonia Hsieh) [108].

As shown in figure 9, water-walking lizards create air cavities as their feet strike the water’s surface and withdraw their feet before the cavities collapse, effectively using a sequential combination of water entry and exit to remain above the surface [39,108–111].

The simple scaling argument of water entry predicts the cavity collapse time and suggests what is the undulation frequency that the animal needs to operate in order to take advantage of drag reduction. Here, we assume that each layer of fluid is independent, and only the radial velocity of the cavity is considered [112]. The time for the cavity to pinch-off is presumably the sum of the time for the plate to reach the depth z and the time for the cavity to collapse to zero radius. When the plate moves at a constant speed, the reach time becomes z/V and the collapse time is $R/v_{r}$ where $v_{r}$ is the radial velocity due to the pressure jump across the interface. From the Bernoulli equation, the inward radial velocity $v_{r}$ is balanced with gravitational potential and surface tension. By minimizing the total time to pinch-off in various depths, one gets the pinch-off height $z_{pinch-off}∼R\;{\text{Fr}}^{1/3}$ and the pinch-off time $t_{pinch-off}∼R/g\;{\text{Fr}}^{-1/6}$.

To maximize the efficiency for locomotion, more drag in the power stroke and less in the recovery are needed. Such asymmetric drag can be achieved if the water-running animal pulls out its limbs before the air cavity collapses as shown in figure 9b. It indicates that the undulation frequency of limbs might be related to the cavity-collapsing time. With the simple allometric assumption (animal’s mass is proportional to the cube of the length), the above calculation on pinch-off time implies that the undulation frequency should be scaled as $f_{\mathrm{foot}}∼1/t_{pinch-off}M^{-2/9}$ to get drag reduction on the recovery stroke. To some extent, this prediction ($f_{\mathrm{foot}}∼M^{-0.222}$) is close to observations in physical experiments mimicking basilisk lizards ($f_{\mathrm{foot}}∼M^{-0.126}$ in [110]). Likewise, such simple scaling can reflect a few key features found in the complicated dynamics exhibited by basilisk lizards.

Similarly, carnivores such as cats and dogs lap water using cyclic water entry and exit processes. Studies have shown that both feline drinking, from domestic cats to lions, and canine drinking exhibit physical mechanisms involving the formation of a slender water column during water exit [6,113]. This pattern demonstrates how fluid mechanics governs drinking behaviour through the coordinated entry and exit of fluid from the water’s surface.

A simple analysis of water exit with constant acceleration can give some useful information to understand animals’ lapping behaviours. As time goes on and the tongue rises, the liquid column is stretched while surface tension plays a role. The time of collapse (or pinch-off time) is predicted in terms of the most unstable wavenumber and growth rate. The final result showed that pinch-off occurs at the plate and at time $t_{pinch-off}∼(t_{c}L/a{)}^{1/3}=t_{c}\;Bo_{a}^{-1/3}$ [42,43], where the capillary timescale $t_{c}$ is defined as $\sqrt{\rho L^{3}/\sigma }$ and the acceleration-based Bond number is defined as $\text{B}{\text{o}}_{a}=\rho aL^{2}/\sigma$. This scaling works quite well with both cat and dog drinking behaviours [43].

The above scaling predicts that the fluid column pinches off near the disk. However, since the fluid is pinned to the rim of the plate or is smoothly connected to the curved tongue surface, pinch-off cannot occur exactly at the surface, but rather at a distance from the surface set by the capillary length $L_{c}$. The entrained volume thus scales as $\pi R^{2}L_{c}$, as in the case of a pendant drop. A pendant drop (i.e. a water volume adhered to the tongue) is determined by inertia and capillary forces during the lapping process. The experiments with a moving object [6,42] show the pinch-off happens close to the plate approximately at a distance of capillary length. Also, by measuring the fluid intake of dog lapping in biological experiments [114], the intake volume per lapping was found to be about 2.5 ml (≡ 2500 mm${,}^{3}$). Based on the calculation above, this result is of the same order of magnitude compared to the prediction of 1800 mm${,}^{3}$ (the estimated intake volume based on R∼ 15 mm is the radius of the touching part of tongue and the capillary length $L_{c}∼$ 2.5 mm).

## 4. Engineering systems

Since there is a vast amount of literature on this topic, I will not go over details, instead provide several review papers and summarize a few key papers.

### 4.1. Water entry in marine engineering

Water entry is important in naval architecture and marine engineering, particularly in the design of ships, submarines and high-speed projectiles. The shape evolution of diving birds, as discussed in §3.1, could provide insights into projectile designs for water entry. When a body enters the water, it experiences rapid deceleration. This deceleration originates from significant fluid forces, including impact force, hydrodynamic drag and hydrostatic pressure as discussed in §2, which must be carefully accounted for in the design of marine vessels to ensure their structural integrity under such loads [115,116]. Detailed mathematical models have been developed for various shapes and different conditions [12,49,117,118].

Hydroelastic coupling between a body entering the water and the surrounding fluid has received a lot of attention from engineers [119–125]. This coupling arises in the water-entry dynamics of flexible bodies, where the structural flexibility of the object interacts with local fluid forces to influence impact dynamics, cavity formation and splash jets [119,120,124]. Understanding these interactions is critical for applications in marine and aerospace systems, where managing hydrodynamic loads is essential. For example, water-entry dynamics are critical in the design of space capsules, which return to Earth by impacting the ocean surface to avoid catastrophic damage [126]. Researchers have focused on how deformation during water entry affects the transient pressure distribution and impact forces and vice versa. Advancements in technology have enabled detailed pressure and force measurements during water entry, providing invaluable data for validating theoretical and computational models [127]. High-speed imaging techniques, advanced pressure sensors and modern computational simulations now allow for precise characterization of the interaction between flexible water-entering bodies and fluid.

### 4.2. Water exit in marine vehicles or projectiles

Water exit is also a crucial phenomenon in marine systems, particularly for vessels, underwater vehicles and other marine structures that transition between underwater and aerial domains [128,129]. The dynamics of animal leaping out of water, as examined in §3.2, share similar dynamics as in engineered water-crossing systems. This process occurs when an object, which was previously submerged, emerges from the water and re-enters the air. Understanding the forces and dynamics involved in water exit is important for designing marine systems that operate efficiently and maintain structural integrity during this transition [130,131]. Like water entry, water exit is governed by the same hydrodynamic forces, but some modifications are needed depending on some details described in §§2 and 3.

When a body exits water, it experiences a complex interplay of hydrodynamic forces that are opposite in the direction to those during water entry [130,132,133] as described earlier. As the body emerges out of water, these drag forces decrease rapidly because air provides significantly less resistance than water. This reduction in resistance allows for a sudden increase in the speed of the object propelled with a constant thrust force as it leaves the water, which must be carefully controlled in engineering designs to prevent damage from excessive acceleration. In marine engineering, the study of water exit is particularly relevant to high-speed systems such as missiles, submarines and launch vehicles that must break through the water surface during operation [134].

### 4.3. Repeated water entry and exit cycles

The ricochet and bouncing of projectiles off a free surface are examples showing both water entry and subsequent water-exit dynamics in a cyclic fashion [135,136]. Similarly, as discussed in §3.3, animal lapping behaviours involve repeated water entry and exit, analogous to engineered systems requiring cyclic interactions with a fluid interface. These dynamics involve complex physical processes, including rapid changes in pressure distribution and momentum transfer between the projectile and the fluid. For example, as a ship moves along a wavy surface, it undergoes repeated impacts and exits, a phenomenon known as ship slamming. These dynamics become increasingly complex when coupled with other factors such as structural elasticity, ship pitching/rolling and angles of incidence [9,137,138]. Understanding ship slamming is crucial in marine engineering to prevent structural damage, protect onboard instruments and minimize discomfort for passengers. Early work by Kaplan explored the physics of ricochet, how the angle of incidence, velocity and projectile shape influence the likelihood of bouncing or penetrating the water surface [139]. The dynamics of skipping stones and other similar projectile motions, emphasizing the role of hydrodynamics, elasticity and/or shape in stabilizing trajectories and prolonging the bouncing sequence [136,140,141]. The work revealed the intricate flow structures around impacting objects that occur during the objects’ interactions with the water surface hydrodynamically [135,136,142].

## 5. Discussion

Understanding the fluid mechanics of water entry and exit is crucial for both biological and engineered systems. While biologists study how animals dive, leap and transition between air and water with certain shapes and kinematics, engineers develop systems that mostly withstand impact forces, minimize drag and optimize performance across the air–water interface. Despite the shared physics, research in biology and engineering has largely progressed in parallel, with limited integration of knowledge between the two disciplines. This review aims to highlight the common physical principles governing water entry and exit across both domains and explore how insights from one field can inform the other.

Nature has evolved efficient mechanisms to overcome challenges that engineers also face in designing water entry and exit systems. For example, the beak shape of plunge-diving birds reduces impact forces, presumably inspiring the design of nose cones for high-speed projectiles [143,144] and space capsules. Likewise, bio-inspired aerial–aquatic drones would draw inspirations from the locomotion of jumping fish and diving birds, enhancing transition efficiency between air and water [145,146]. Amphibious robots, which must navigate repeated water entry and exit, would benefit from studying how water-walking animals like basilisks exploit surface tension and stride frequency to remain on the surface. Further research should continue to explore the connections between animal behaviours and engineering solutions, with the goal of developing bio-inspired technologies that enhance our ability to navigate and interact with aquatic environments.

## Data Availability

All data are available at OSF [147].

## References

[1] Louf JF, Chang B, Eshraghi J, Mituniewicz A, Vlachos PP, Jung S. 2018 Cavity ripple dynamics after pinch-off. J. Fluid Mech. **850**, 611–623. (10.1017/jfm.2018.459)

[2] Kim SJ, Hasanyan J, Gemmell BJ, Lee S, Jung S. 2015 Dynamic criteria of plankton jumping out of water. J. R. Soc. Interface **12**, 20150582. (10.1098/rsif.2015.0582) 26468066 PMC4614495

[3] Hayward R. 2025 Pexels. See https://www.pexels.com/video/speed-boats-racing-in-the-ocean-5340077/.

[4] Chang B, Croson M, Straker L, Gart S, Dove C, Gerwin J, Jung S. 2016 How seabirds plunge-dive without injuries. Proc. Natl Acad. Sci. USA **113**, 12006–12011. (10.1073/pnas.1608628113) 27702905 PMC5087068

[5] Chang B, Myeong J, Virot E, Clanet C, Kim HY, Jung S. 2019 Jumping dynamics of aquatic animals. J. R. Soc. Interface **16**, 20190014. (10.1098/rsif.2019.0014) 30836892 PMC6451394

[6] Gart S, Socha JJ, Vlachos PP, Jung S. 2015 Dogs lap using acceleration-driven open pumping. Proc. Natl Acad. Sci. USA **112**, 15798–15802. (10.1073/pnas.1514842112) 26668382 PMC4703018

[7] Wagner H. 1932 Phenomena associated with impacts and sliding on liquid surfaces. J. Appl. Math. Mech. **12**, 193–215. (10.1002/zamm.19320120402)

[8] Kármán T. 1929 The impact on seaplane floats during landing. (NACA Technical Note, no. 321).

[9] Faltinsen OM. 2006 Hydrodynamics of high-speed marine vehicles. New York, NY: Cambridge University Press.

[10] Lamb H. 1945 Hydrodynamics dover. vol. 43. New York, NY: Dover Publications.

[11] Bush JWM, Hu DL. 2006 Walking on water: biolocomotion at the interface. Annu. Rev. Fluid Mech. **38**, 339–369. (10.1146/annurev.fluid.38.050304.092157)

[12] Truscott TT, Epps BP, Belden J. 2014 Water entry of projectiles. Annu. Rev. Fluid Mech. **46**, 355–378. (10.1146/annurev-fluid-011212-140753)

[13] Eshraghi J, Jung S, Vlachos PP. 2020 To seal or not to seal: the closure dynamics of a splash curtain. Phys. Rev. Fluids **5**, 104001. (10.1103/physrevfluids.5.104001)

[14] White FM. 2015 Fluid mechanics. New York, NY: McGraw-Hill Education.

[15] Batchelor GK. 1967 An introduction to fluid dynamics. Cambridge, UK: Cambridge University Press.

[16] Hoerner SF. 1965 Fluid dynamic drag: practical information on aerodynamic drag and hydrodynamic resistance. Bakersfield, CA: Hoerner Fluid Dynamics.

[17] Huang KN, Xiao W, Yao XL, Liu JL. 2023 Numerical investigation on cavity dynamics of water-entry bodies with different shape parameters. Phys. Fluids **35**, 062115. (10.1063/5.0153088)

[18] Weast RC. 1986 CRC handbook of chemistry and physics. Boca Raton, FL: CRC Press.

[19] Prezioso D, Di domenico D, Pane M, Ciccarelli D, D’errico G. 2019 Ion specificity in determining physico-chemical properties of drinking water. Food Sci. Technol. **39**, 485–490. (10.1590/fst.34717)

[20] Veldhuis C, Biesheuvel A, van Wijngaarden L. 2008 Shape oscillations on bubbles rising in clean and in tap water. Phys. Fluids **20**, 040705. (10.1063/1.2911042)

[21] Nayar KG, Panchanathan D, McKinley GH, Lienhard JH V. 2014 Surface tension of seawater. J. Phys. Chem. Ref. Data **43**, 043103. (10.1063/1.4899037)

[22] Lide DR. 2005 CRC handbook of chemistry and physics, 86th edn. Boca Raton, FL: CRC Press.

[23] Takamura K, Fischer H, Morrow NR. 2012 Physical properties of aqueous glycerol solutions. J. Pet. Sci. Eng **98**, 50–60. (10.1016/j.petrol.2012.09.003)

[24] Mendonça CGD, Raetano CG, Mendonça CG. 2007 Surface tension of mineral oils and vegetable oils. Eng. Agric. **27**, 16–23.

[25] Kim S, Cho YI, Kensey KR, Pellizzari RO, Stark PRH. 2000 A scanning dual-capillary-tube viscometer. Rev. Sci. Instrum. **71**, 3188–3192. (10.1063/1.1305513)

[26] Melo-Espinosa EA, Sánchez-Borroto Y, Errasti M, Piloto-Rodríguez R, Sierens R, Roger-Riba J, Christopher-Hansen A. 2014 Surface tension prediction of vegetable oils using artificial neural networks and multiple linear regression. Energy Proc. **57**, 886–895. (10.1016/j.egypro.2014.10.298)

[27] Schaschke CJ, Abid S, Heslop MJ. 2007 High-pressure viscosity measurement of fatty acids and oils. High Press. Res. **27**, 33–37. (10.1080/08957950601090097)

[28] Schmidt-Nielsen K. 1984 Scaling: why is animal size so important?. Cambridge, UK: Cambridge University Press. (10.1017/CBO9781139167826)

[29] Bainbridge R. 1958 The speed of swimming of fish as related to size and to the frequency and amplitude of the tail beat. J. Exp. Biol. **35**, 109–133. (10.1242/jeb.35.1.109)

[30] Gazzola M, Argentina M, Mahadevan L. 2014 Scaling macroscopic aquatic locomotion. Nat. Phys. **10**, 758–761. (10.1038/nphys3078)

[31] Jung S. 2021 Swimming, flying, and diving behaviors from a unified 2D potential model. Sci. Rep. **11**, 15984. (10.1038/s41598-021-94829-7) 34362958 PMC8346475

[32] Bale R, Hao M, Bhalla APS, Patankar NA. 2014 Energy efficiency and allometry of movement of swimming and flying animals. Proc. Natl Acad. Sci. USA **111**, 7517–7521. (10.1073/pnas.1310544111) 24821764 PMC4040623

[33] Triantafyllou GS, Triantafyllou MS, Grosenbaugh MA. 1993 Optimal thrust development in oscillating foils with application to fish propulsion. J. Fluids Struct. **7**, 205–224. (10.1006/jfls.1993.1012)

[34] Triantafyllou MS, Triantafyllou GS, Yue DKP. 2000 Hydrodynamics of fishlike swimming. Annu. Rev. Fluid Mech. **32**, 33–53. (10.1146/annurev.fluid.32.1.33)

[35] Vogel S. 2008 Modes and scaling in aquatic locomotion. Integr. Comp. Biol. **48**, 702–712. (10.1093/icb/icn014) 21669826

[36] Eloy C. 2012 Optimal Strouhal number for swimming animals. J. Fluids Struct. **30**, 205. (10.1016/j.jfluidstructs.2012.02.008)

[37] Hirt MR, Jetz W, Rall B, Brose U. 2017 A general scaling law reveals why the largest animals are not the fastest. Nat. Ecol. Evol. **1**, 1116–1122. (10.1038/s41559-017-0241-4) 29046579

[38] Gans C. 1976 The process of skittering in frogs. Ann. Zool **12**, 37–40.

[39] Glasheen JW, Mcmahon TA. 1996 Size-dependence of water-running ability in basilisk lizards (Basiliscus basiliscus). J. Exp. Biol. **199**, 2611–2618. (10.1242/jeb.199.12.2611) 9320547

[40] Clanet C, Hersen F, Bocquet L. 2004 Secrets of successful stone-skipping. Nature **427**, 29–29. (10.1038/427029a) 14702075

[41] Rosellini L, Hersen F, Clanet C, Bocquet L. 2005 Skipping stones. J. Fluid Mech. **543**, 137. (10.1017/s0022112005006373)

[42] Kim SJ, Kim S, Jung S. 2018 Extremes of the pinch-off location and time in a liquid column by an accelerating solid sphere. Phys. Rev. Fluids **3**, 084001. (10.1103/physrevfluids.3.084001)

[43] Jung S. 2021 Pinch-off dynamics to describe animal lapping. Phys. Rev. Fluids **6**, 073102. (10.1103/PhysRevFluids.00.003100)

[44] Taylor GK, Nudds RL, Thomas ALR. 2003 Flying and swimming animals cruise at a Strouhal number tuned for high power efficiency. Nature **425**, 707–711. (10.1038/nature02000) 14562101

[45] Williamson CH, Govardhan R. 2004 Vortex-induced vibrations. Annu. Rev. Fluid Mech **36**, 413–455. (10.1146/annurev.fluid.36.050802.122128)

[46] Bearman P. 1984 Vortex shedding from oscillating bluff bodies. Annu. Rev. Fluid Mech. **16**, 195–222. (10.1146/annurev.fluid.16.1.195)

[47] Livezey BC, Humphrey PS. 1983 Mechanics of steaming in steamer-ducks. Auk **100**, 485–488. (10.1093/auk/100.2.485)

[48] Aigeldinger TL, Fish FE. 1995 Hydroplaning by ducklings: overcoming limitations to swimming at the water surface. J. Exp. Biol. **198**, 1567–1574. (10.1242/jeb.198.7.1567) 9319469

[49] Seddon CM, Moatamedi M. 2006 Review of water entry with applications to aerospace structures. Int. J. Impact Eng. **32**, 1045–1067. (10.1016/j.ijimpeng.2004.09.002)

[50] Allen WH. 1995 Animals and their models do. BioScience **45**, 381–383.

[51] Katzir G, Camhi JM. 1993 Escape response of black mollies (Poecilia sphenops) to predatory dives of a pied kingfisher (Ceryle rudis). Copeia **1993**, 549. (10.2307/1447160)

[52] Weiss T, Gillis GB, Van Mullekom J, Socha JJ. 2024 Skittering locomotion in cricket frogs: a form of porpoising. J. Exp. Biol. **227**, 249403. (10.1242/jeb.249403) 39415737

[53] Federation NW. Grizzly bear. See https://www.nwf.org/Educational-Resources/Wildlife-Guide/Mammals/Grizzly-Bear.

[54] Mattson DJ. 2003 Foot loadings and pad and track widths of Yellowstone grizzly bears. West. N. Am. Nat **63**, 72–79.

[55] Wright N. Ospreys: a unique bird of prey. See https://www.nwrafting.com/articles/ospreys-a643unique-bird-of-prey.

[56] Pandey A, Yuk J, Chang B, Fish FE, Jung S. 2022 Slamming dynamics of diving and its implications for diving-related injuries. Sci. Adv. **8**, eabo5888. (10.1126/sciadv.abo5888) 35895822 PMC9328685

[57] Olsen AM. 2017 Feeding ecology is the primary driver of beak shape diversification in waterfowl. Funct. Ecol. **31**, 1985–1995. (10.1111/1365-2435.12890)

[58] Bout RG, Zweers GA. 2001 The role of cranial kinesis in birds. Comp. Biochem. Physiol. A **131**, 197–205. (10.1016/S1095-6433(01)00470-6) 11733177

[59] Zusi R. 1993 patterns of structural and systematic diversity. In The skull, vol. 2 (eds J Hanken, BK Hall). Chicago, IL: University of Chicago Press.

[60] Owre OT. 1967 Adaptations for locomotion and feeding in the anhinga and the double-crested cormorant. Ornithol. Monogr. 1–138. (10.2307/40166666)

[61] Bhar K, Chang B, Virot E, Straker L, Kang H, Paris R, Clanet C, Jung S. 2019 How localized force spreads on elastic contour feathers. J. R. Soc. Interface **16**, 20190267. (10.1098/rsif.2019.0267) 31744417 PMC6893494

[62] Yang S hui, Xu Y chun, Zhang D wei. 2006 Morphological basis for the waterproof characteristic of bird plumage. J. For. Res. **17**, 163–166. (10.1007/s11676-006-0039-8)

[63] Debenedetti F, Jung S. 2024 Effect of feathers on drag in plunge‐diving birds. Ann. N. Y. Acad. Sci. **1537**, 74–81. (10.1111/nyas.15181) 38963660

[64] Jarvis ED, Mirarab S, Aberer AJ, Li B, Houde P, Li C, Ho SY. 2014 Whole-genome analyses resolve early branches in the tree of life of modern birds. Science **346**, 1320–1331. (10.1126/science.1253451) 25504713 PMC4405904

[65] Dunn EK. 1972 Effect of age on the fishing ability of sandwich terns Sterna sandvicensis. Ibis **114**, 360–366. (10.1111/j.1474-919x.1972.tb00833.x)

[66] Ropert‐Coudert Y, Grémillet D, Ryan P, Kato A, Naito Y, Le Maho Y. 2004 Between air and water: the plunge dive of the Cape gannet Morus capensis. Ibis **146**, 281–290. (10.1111/j.1474-919x.2003.00250.x)

[67] Brierley AS, Fernandes PG. 2001 Diving depths of northern gannets: acoustic observations of Sula bassana from an autonomous underwater vehicle. Auk **118**, 529. (10.1642/0004-8038(2001)118[0529:ddonga]2.0.co;2)

[68] Huin N. 1994 Diving depths of white-chinned petrels. Condor **96**, 1111–1113. (10.2307/1369125)

[69] Katzir G, Camhi JM. 1993 Escape response of black mollies (Poecilia sphenops) to predatory dives of a pied kingfisher (Ceryle rudis). Copeia **2**, 549. (10.2307/1447160)

[70] Machovsky Capuska G, Vaughn R, Würsig B, Katzir G, Raubenheimer D. 2011 Dive strategies and foraging effort in the Australasian gannet Morus serrator revealed by underwater videography. Mar. Ecol. Prog. Ser. **442**, 255–261. (10.3354/meps09458)

[71] Machovsky-Capuska GE, Vaughn-Hirshorn RL, Würsig B, Raubenheimer D. 2013 Can gannets (Morus serrator) select their diving profile prior to submergence? Notornis **60**, 255. (10.63172//592582tfraxq)

[72] Ropert‐Coudert Y, Daunt F, Kato A, Ryan PG, Lewis S, Kobayashi K, Mori Y, Grémillet D, Wanless S. 2009 Underwater wingbeats extend depth and duration of plunge dives in northern gannets Morus bassanus. J. Avian Biol. **40**, 380–387. (10.1111/j.1600-048x.2008.04592.x)

[73] Shoop WL, Tilson E. 2022 Plunge diving by brown pelicans resembles a split-S turn. J. Field Ornithol. **93**. (10.5751/JFO-00064-930102)

[74] Lee DN, Reddish PE. 1981 Plummeting gannets: a paradigm of ecological optics. Nature **293**, 293–294. (10.1038/293293a0)

[75] Halsey LG, Butler PJ, Blackburn TM. 2006 A phylogenetic analysis of the allometry of diving. Am. Nat. **167**, 276–287. (10.1086/499439) 16670986

[76] Eliason CM, Straker L, Jung S, Hackett SJ. 2020 Morphological innovation and biomechanical diversity in plunge‐diving birds. Evolution **74**, 1514–1524. (10.1111/evo.14024) 32452015

[77] Sharker SI *et al*. 2019 Water entry impact dynamics of diving birds. Bioinspir. Biomim **14**, 056013. (10.1088/1748-3190/ab38cc) 31387087

[78] Crandell KE, Howe RO, Falkingham PL. 2019 Repeated evolution of drag reduction at the air–water interface in diving kingfishers. J. R. Soc. Interface **16**, 20190125. (10.1098/rsif.2019.0125) 31088257 PMC6544885

[79] Butler PJ, Jones DR. 1997 Physiology of diving of birds and mammals. Physiol. Rev. **77**, 837–899. (10.1152/physrev.1997.77.3.837) 9234967

[80] Zimmerman S, Ceballes S, Taylor G, Chang B, Jung S, Abdelkefi A. 2019 Nonlinear modeling and experimental verification of gannet-inspired beam systems during diving. Bioinspir. Biomim. **14**, 026002. (10.1088/1748-3190/aaf98c) 30562725

[81] Zimmerman S, Abdelkefi A. 2020 Enhanced design considerations on the buckling and dynamics of gannet-inspired systems during water entry. Bioinspir. Biomim. **16**, 026011. (10.1088/1748-3190/abc468) 33096538

[82] Zimmerman S, Abdelkefi A. 2020 Investigations on the buckling and dynamics of diving-inspired systems when entering water. Bioinspir. Biomim. **15**, 036015. (10.1088/1748-3190/ab76d8) 32066135

[83] Wells KD. 2019 The ecology and behavior of amphibians. Chicago, IL: University of Chicago Press.

[84] Gregorio E, Balaras E, Leftwich MC. 2023 Air cavity deformation by single jointed diver model entry bodies. Exp. Fluids **64**, 168. (10.1007/s00348-023-03712-w)

[85] Rubin BD. 1999 The basics of competitive diving and its injuries. Clin. Sports Med. **18**, 293–303. (10.1016/s0278-5919(05)70145-9) 10230565

[86] Gemmell BJ, Jiang H, Strickler JR, Buskey EJ. 2012 Plankton reach new heights in effort to avoid predators. Proc. R. Soc. B **279**, 2786–2792. (10.1098/rspb.2012.0163) PMC3367780 22438496

[87] Kim A, Lee C, Kim H, Kim J. 2015 Simple approach to superhydrophobic nanostructured Al for practical antifrosting application based on enhanced self-propelled jumping droplets. ACS Appl. Mater. Interfaces **7**, 7206–7213. (10.1021/acsami.5b00292) 25782028

[88] Lowry D, Wintzer AP, Matott MP, Whitenack LB, Huber DR, Dean M, Motta PJ. 2005 Aerial and aquatic feeding in the silver arawana, Osteoglossum bicirrhosum. Environ. Biol. Fishes **73**, 453–462. (10.1007/s10641-005-3214-4)

[89] Nauwelaerts S, Scholliers J, Aerts P. 2004 A functional analysis of how frogs jump out of water. Biol. J. Linn. Soc. **83**, 413–420. (10.1111/j.1095-8312.2004.00403.x)

[90] Vetter B, Casper A, Mensinger A. 2017 Characterization and management implications of silver carp (Hypophthalmichthys molitrix) jumping behavior in response to motorized watercraft. Manag. Biol. Invasions **8**, 113–124. (10.3391/mbi.2017.8.1.11)

[91] Lauritzen DV, Hertel F, Gordon MS. 2005 A kinematic examination of wild sockeye salmon jumping up natural waterfalls. J. Fish Biol. **67**, 1010–1020. (10.1111/j.0022-1112.2005.00799.x)

[92] Kondratieff MC, Myrick CA. 2006 How high can brook trout jump? A laboratory evaluation of brook trout jumping performance. Trans. Am. Fish. Soc. **135**, 361–370. (10.1577/t04-210.1)

[93] Soares D, Bierman HS. 2013 Aerial jumping in the Trinidadian guppy (Poecilia reticulata). PLoS One **8**, e61617. (10.1371/journal.pone.0061617) 23613883 PMC3629028

[94] Smith HK. 1998 Applied physiology of water polo. Sports Med. **26**, 317–334. (10.2165/00007256-199826050-00003) 9858395

[95] McCluskey L, Lynskey S, Leung CK, Woodhouse D, Briffa K, Hopper D. 2010 Throwing velocity and jump height in female water polo players: performance predictors. J. Sci. Med. Sport **13**, 236–240. (10.1016/j.jsams.2009.02.008) 19442582

[96] Sato K, Ponganis PJ, Habara Y, Naito Y. 2005 Emperor penguins adjust swim speed according to the above-water height of ice holes through which they exit. J. Exp. Biol. **208**, 2549–2554. (10.1242/jeb.01665) 15961741

[97] Davenport J, Hughes R, Shorten M, Larsen P. 2011 Drag reduction by air release promotes fast ascent in jumping emperor penguins—a novel hypothesis. Mar. Ecol. Prog. Ser. **430**, 171–182. (10.3354/meps08868)

[98] Medeiros A, Ari C, Monteiro-Filho E. 2021 Environmental factors involved in breaching behavior of manta rays in estuarine waters. Mar. Ecol. Prog. Ser. **674**, 203–219. (10.3354/meps13815)

[99] Klimley AP, Curtis TH, Johnston EM, Kock A, Stevens GMW. 2024 A review of elasmobranch breaching behavior: why do sharks and rays propel themselves out of the water into the air? Environ. Biol. Fishes **108**, 441–481. (10.1007/s10641-024-01584-5)

[100] Au D, Weihs D. 1980 At high speeds dolphins save energy by leaping. Nature **284**, 548–550. (10.1038/284548a0)

[101] Blake RW. 1983 Energetics of leaping in dolphins and other aquatic animals. J. Mar. Biol. Ass. **63**, 61–70. (10.1017/S0025315400049808)

[102] Hui CA. 1989 Surfacing behavior and ventilation in free-ranging dolphins. J. Mammal. **70**, 833–835. (10.2307/1381722)

[103] Weihs D. 2002 Dynamics of dolphin porpoising revisited. Integr. Comp. Biol. **42**, 1071–1078. (10.1093/icb/42.5.1071) 21680390

[104] Fish FE, Nicastro AJ, Weihs D. 2006 Dynamics of the aerial maneuvers of spinner dolphins. J. Exp. Biol. **209**, 590–598. (10.1242/jeb.02034) 16449554

[105] Brunnschweiler JM. 2005 Water-escape velocities in jumping blacktip sharks. J. R. Soc. Interface **2**, 389–391. (10.1098/rsif.2005.0047) 16849197 PMC1578268

[106] Whitehead H. 1985 Humpback whale breaching. Investig. Cetacea **17**, 117–156.

[107] Shih AM, Mendelson L, Techet AH. 2017 Archer fish jumping prey capture: kinematics and hydrodynamics. J. Exp. Biol. **220**, 1411–1422. (10.1242/jeb.145623) 28424312

[108] Hsieh ST. 2003 Three-dimensional hindlimb kinematics of water running in the plumed basilisk lizard (Basiliscus plumifrons). J. Exp. Biol. **206**, 4363–4377. (10.1242/jeb.00679) 14581605

[109] Glasheen JW, McMahon TA. 1996 Vertical water entry of disks at low Froude numbers. Phys. Fluids **8**, 2078–2083. (10.1063/1.869010)

[110] Glasheen JW, McMahon TA. 1996 A hydrodynamic model of locomotion in the basilisk lizard. Nature **380**, 340–342. (10.1038/380340a0)

[111] Aristoff JM, Stocker R, Reis PM, Jung S. 2011 A unifying physical perspective. Commun. Integr. Biol **4**, 213–215. (10.4161/cib.4.2.14493) 21655444 PMC3104583

[112] Duclaux V, Caillé F, Duez C, Ybert C, Bocquet L, Clanet C. 2007 Dynamics of transient cavities. J. Fluid Mech. **591**, 1–19. (10.1017/s0022112007007343)

[113] Reis PM, Jung S, Aristoff JM, Stocker R. 2010 How cats lap: water uptake by Felis catus. Science **330**, 1231–1234. (10.1126/science.1195421) 21071630

[114] Adolph EF. 1938 Measurements of water drinking in dogs. Am. J. Physiol. Leg. Content **125**, 75–86. (10.1152/ajplegacy.1938.125.1.75)

[115] Houlberg K, Wickenden J, Freshwater D. 2019 Five centuries of medical contributions from the Royal Navy. Clin. Med. **19**, 22. (10.7861/clinmedicine.19-1-22) PMC6399651 30651240

[116] Mouritz AP, Gellert E, Burchill P, Challis K. 2001 Review of advanced composite structures for naval ships and submarines. Compos. Struct. **53**, 21–42. (10.1016/s0263-8223(00)00175-6)

[117] Abrate S. 2011 Hull slamming. Appl. Mech. Rev. **64**, 060803. (10.1115/1.4023571)

[118] Mackey A. 1979 A mathematical model of water entry. Admiralty Underwater Weapons Establishment: Portland, UK.(AUWE Technical Note, no. 636/79).

[119] Ren Z, Wang Z, Stern F, Judge C, Ikeda-Gilbert C. 2019 Vertical water entry of a flexible wedge into calm water: a fluid-structure interaction experiment. J. Ship Res. **63**, 41–55. (10.5957/josr.09180087)

[120] Tavakoli S, Mikkola T, Hirdaris S. 2023 A fluid–solid momentum exchange method for the prediction of hydroelastic responses of flexible water entry problems. J. Fluid Mech. **965**, 386. (10.1017/jfm.2023.386)

[121] Shi Y, Pan G, Yim SC, Yan G, Zhang D. 2019 Numerical investigation of hydroelastic water-entry impact dynamics of AUVs. J. Fluids Struct. **91**, 102760. (10.1016/j.jfluidstructs.2019.102760)

[122] Yu C *et al*. 2019 Managing nitrogen to restore water quality in China. Nature **567**, 516–520. (10.1038/s41586-019-1001-1) 30818324

[123] Yang L, Sun T zhi, Wei Y jie, Wang C, Xia W xue, Wang Z lu. 2021 Hydroelastic analysis of water entry of deformable spheres. J. Hydrodyn. **33**, 821–832. (10.1007/s42241-021-0065-1)

[124] Shams A, Zhao S, Porfiri M. 2017 Hydroelastic slamming of flexible wedges: modeling and experiments from water entry to exit. Phys. Fluids **29**, 037107. (10.1063/1.4978631)

[125] Panciroli R, Abrate S, Minak G, Zucchelli A. 2012 Hydroelasticity in water-entry problems: comparison between experimental and SPH results. Compos. Struct. **94**, 532–539. (10.1016/j.compstruct.2011.08.016)

[126] Cappelli AP, Salzman RN, Wilkinson JPD. 1967 Study of Apollo water impact. Vol. 3. Dynamic response of shells of revolution during vertical impact into water: hydroelastic interaction. Final report no. NASA-CR-92021, SID-67-498.

[127] Jain U, Novaković V, Bogaert H, van der Meer D. 2022 On wedge-slamming pressures. J. Fluid Mech. **934**, A27. (10.1017/jfm.2021.1129)

[128] Chu X, Yan K, Wang Z, Zhang K, Feng G, Chen W. 2010 Numerical simulation of water-exit of a cylinder with cavities. J. Hydrodyn. **22**, 834–838. (10.1016/S1001-6058(10)60045-5)

[129] Shi H, Chen B, Wang Y. 2016 Experimental and numerical study of oblique water exit in free surface penetration by a blunt body’s supercavity. Shiyan Liuti Lixue/.Exp. Fluid Mech. **30**, 29–35. (10.11729/syltlx20150154)

[130] Guzel B, Korkmaz FC. 2016 Experimental investigation of water exit under hydrophobic effects. In ASME 2016 35th Int. Conf. on Ocean, Offshore and Arctic Engineering, Busan, South Korea, 19–24 June 2016, paper V007T06A063. (10.1115/OMAE2016-54636)

[131] Zhang C. 2015 Dynamics modeling and simulation of water-exit course of small submarine-launched missile under wave disturbance. J. Natl Univ. Def. Technol. **37**, 91–95. (10.11887/j.cn.201506018)

[132] Moyo S, Greenhow M. 2000 Free motion of a cylinder moving below and through a free surface. Appl. Ocean Res. **22**, 31–44. (10.1016/s0141-1187(99)00024-3)

[133] Zan Y, Qi B, Ding S, Guo R, Wang Y, Li B. 2023 Experimental and numerical investigation of cavity structure forced water exit from calm water at constant lifting velocity. J. Mar. Sci. Eng. **11**, 274. (10.3390/jmse11020274)

[134] Yang J, Feng J, Li Y, Liu A, Hu J, Ma Z. 2017 Water-exit process modeling and added-mass calculation of the submarine-launched missile. Pol. Marit. Res. **24**, 152–164. (10.1515/pomr-2017-0118)

[135] Clanet C, Héraud P, Searby G. 2004 On the motion of bubbles in vertical tubes of arbitrary cross-sections: some complements to the Dumitrescu–Taylor problem. J. Fluid Mech. **519**, 359–376. (10.1017/s0022112004001296)

[136] Belden J, Hurd RC, Jandron MA, Bower AF, Truscott TT. 2016 Elastic spheres can walk on water. Nat. Commun. **7**, 10551. (10.1038/ncomms10551) 26842860 PMC4743002

[137] Kim DJ, Vorus W, Troesch A, Gollwitzer R. 1996 Coupled hydrodynamic impact and elastic response. In Proc. 21st Symp. on Naval Hydrodynamics. Washington, DC: National Academies Press.

[138] Antolik JT, Belden JL, Speirs NB, Harris DM. 2023 Slamming forces during water entry of a simple harmonic oscillator. J. Fluid Mech. **974**, 820. (10.1017/jfm.2023.820)

[139] Kaplan P. 1987 Analysis and prediction of flat bottom slamming impact of advanced marine vehicles in waves. Int. Shipbuild. Prog. **34**, 44–53. (10.3233/isp-1987-3439101)

[140] Tassin A, Piro DJ, Korobkin AA, Maki KJ, Cooker MJ. 2013 Two-dimensional water entry and exit of a body whose shape varies in time. J. Fluids Struct. **40**, 317–336. (10.1016/j.jfluidstructs.2013.05.002)

[141] Hurd RC, Belden J, Bower AF, Holekamp S, Jandron MA, Truscott TT. 2019 Water walking as a new mode of free surface skipping. Sci. Rep. **9**, 6042. (10.1038/s41598-019-42453-x) 30988350 PMC6465409

[142] Del Buono A, Bernardini G, Tassin A, Iafrati A. 2021 Water entry and exit of 2D and axisymmetric bodies. J. Fluids Struct. **103**, 103269. (10.1016/j.jfluidstructs.2021.103269)

[143] Murphy CT, Müller R, Jung S. 2022 Sample digitization techniques for bio-inspired engineering. In Biomimicry for materials, design and habitats (eds M Eggermont, V Shyam, AF Hepp), pp. 215–246. Amsterdam, The Netherlands: Elsevier. (10.1016/b978-0-12-821053-6.00012-6)

[144] Stephen EJ, McLaughlin TE, Bixler B, Dickinson B, Turner J. 2019 Investigation of nose cone enhancement to improve the effectiveness of an articulating nose cone on a subsonic missile. In AIAA Aviation 2019 Forum Dallas, TX, USA, 17–21 June 2019, paper AIAA2019-3165. (10.2514/6.2019-3165)

[145] Sun Y, Liu X, Cao K, Shen H, Li Q, Chen G, Xu J, Ji A. 2023 Design and theoretical research on aerial-aquatic vehicles: a review. J. Bionic Eng. **20**, 2512–2541. (10.1007/s42235-023-00418-x)

[146] Zufferey R, Siddall R, Armanini SF, Kovac M. 2022 Between sea and sky: aerial aquatic locomotion in miniature robots. Cham, Switzerland: Springer.

[147] Jung S. 2025 Water entry and exit review. OSF. (10.17605/OSF.IO/AT9KM) PMC12082843 40386282

